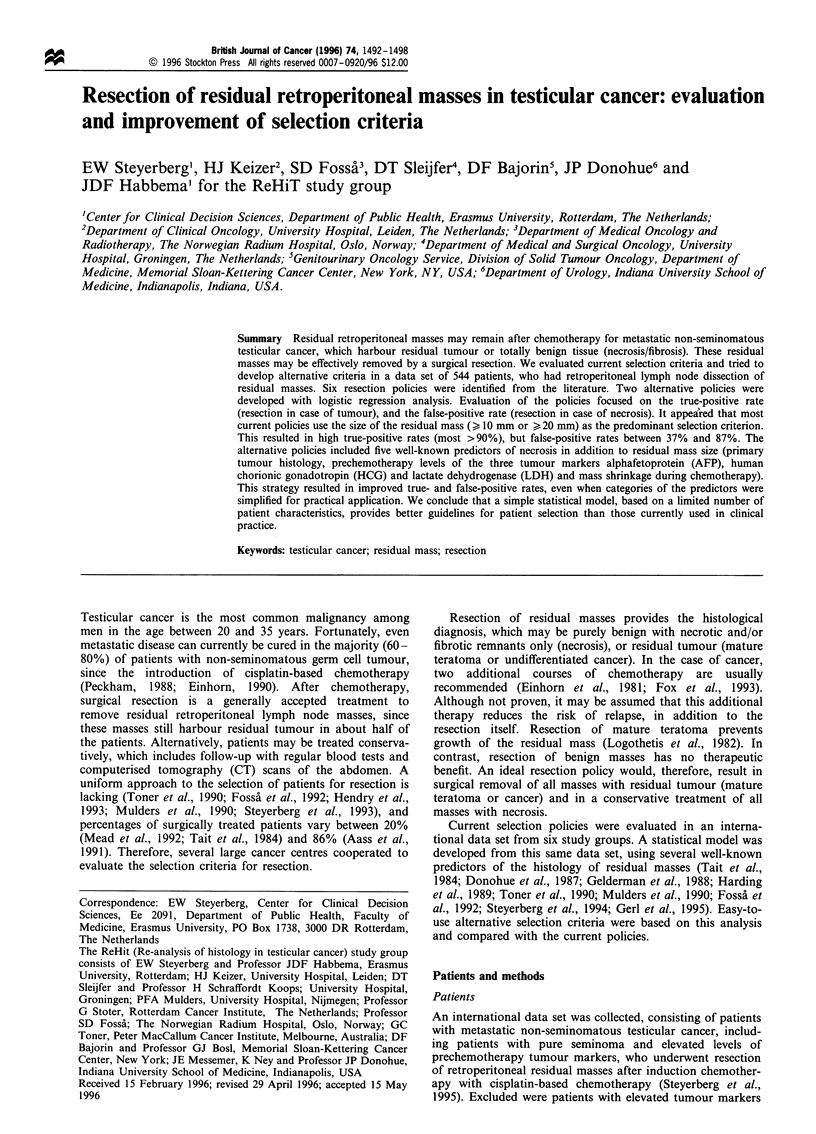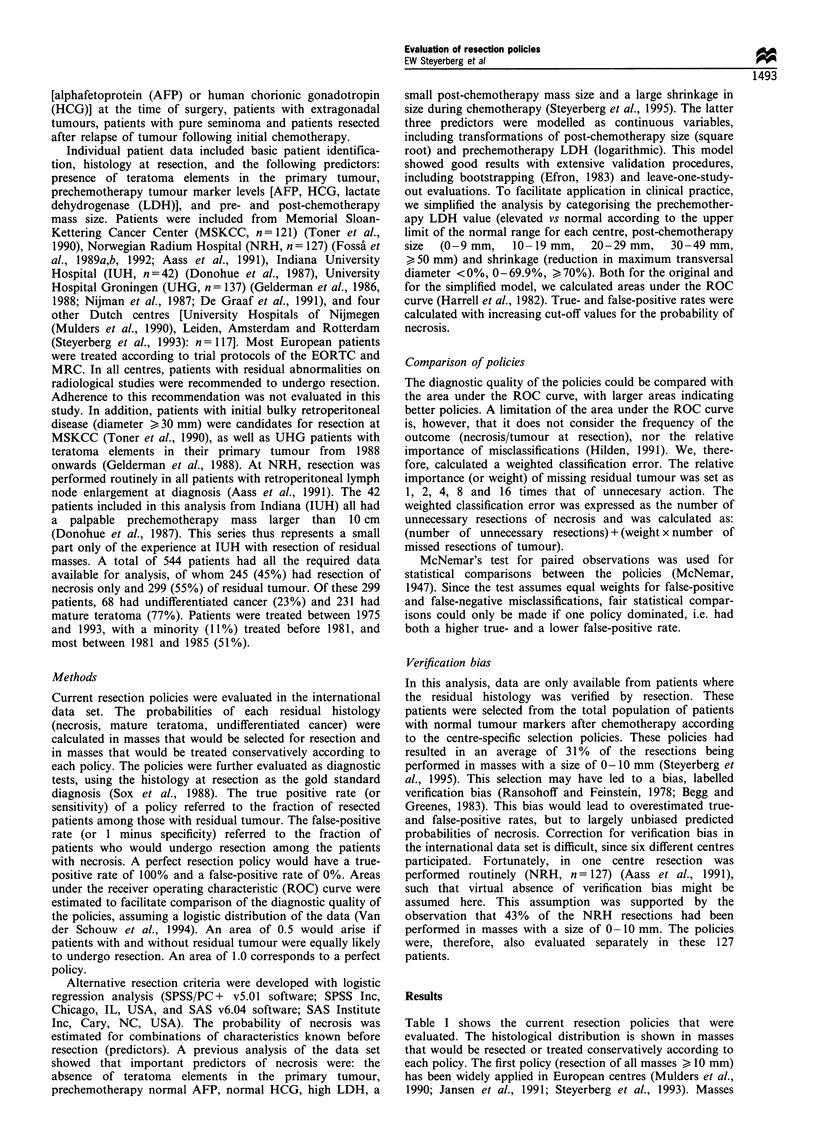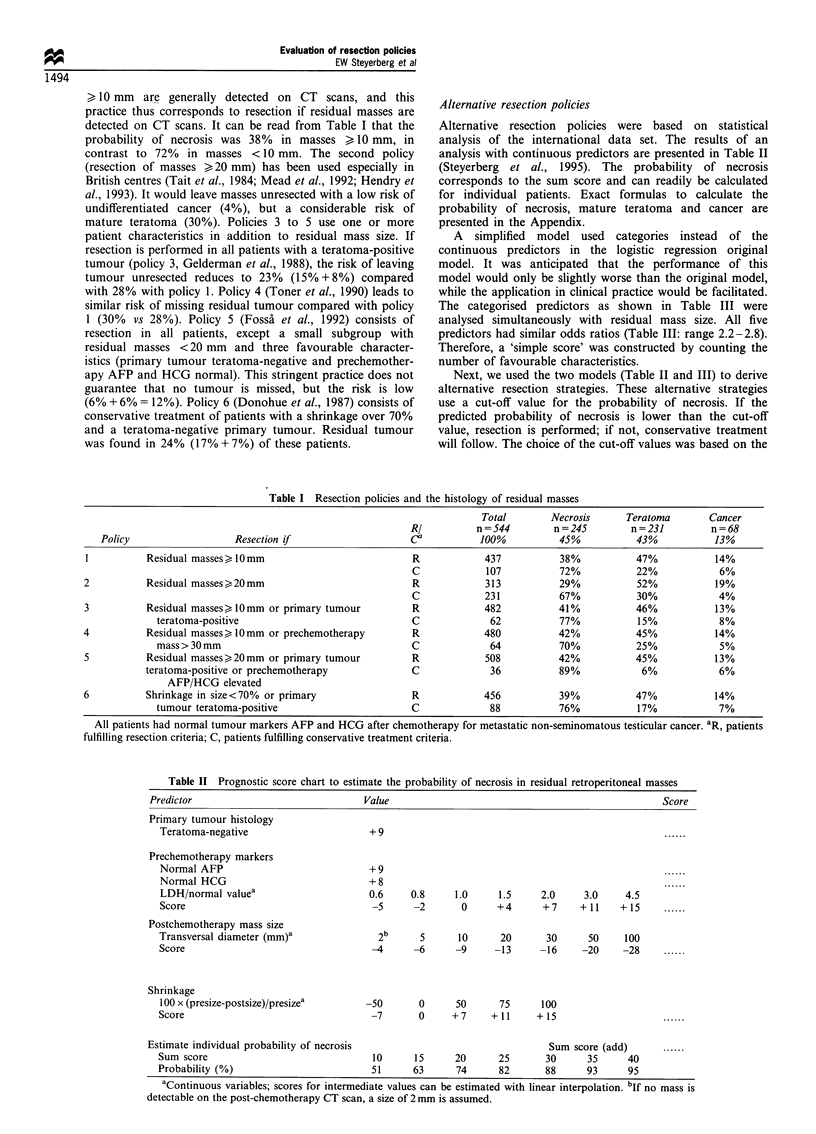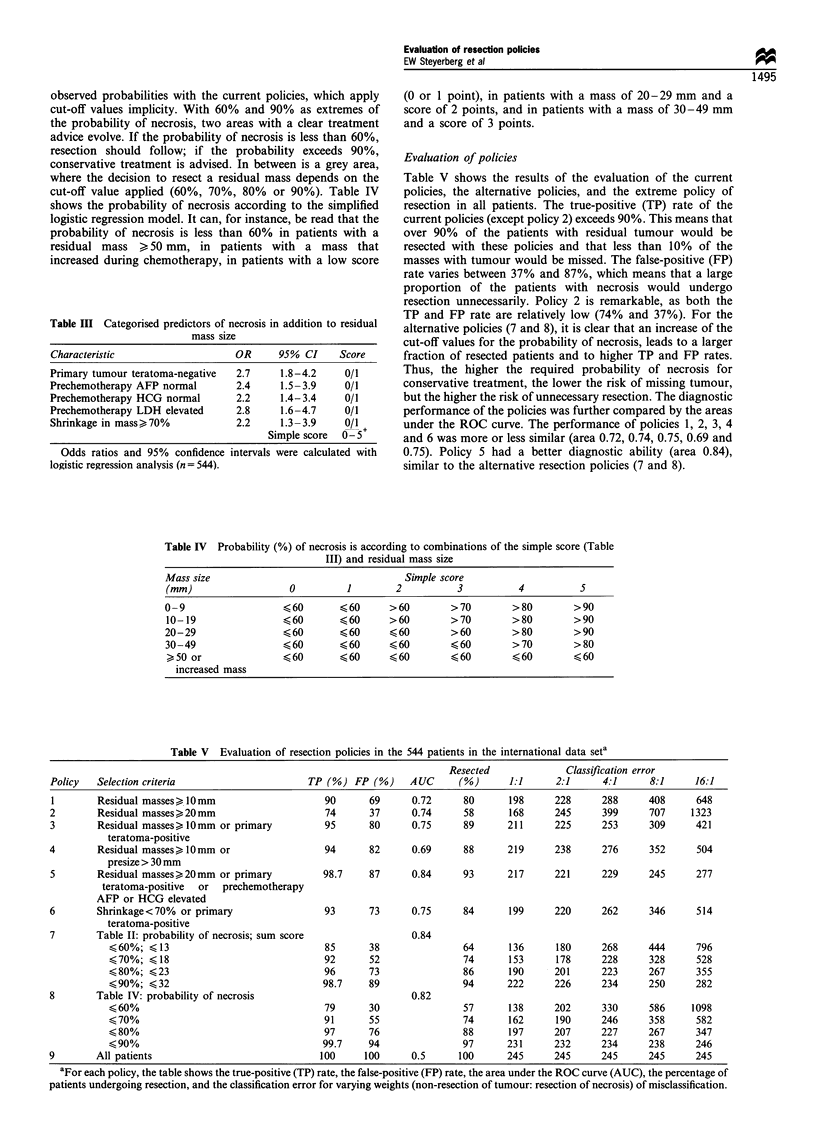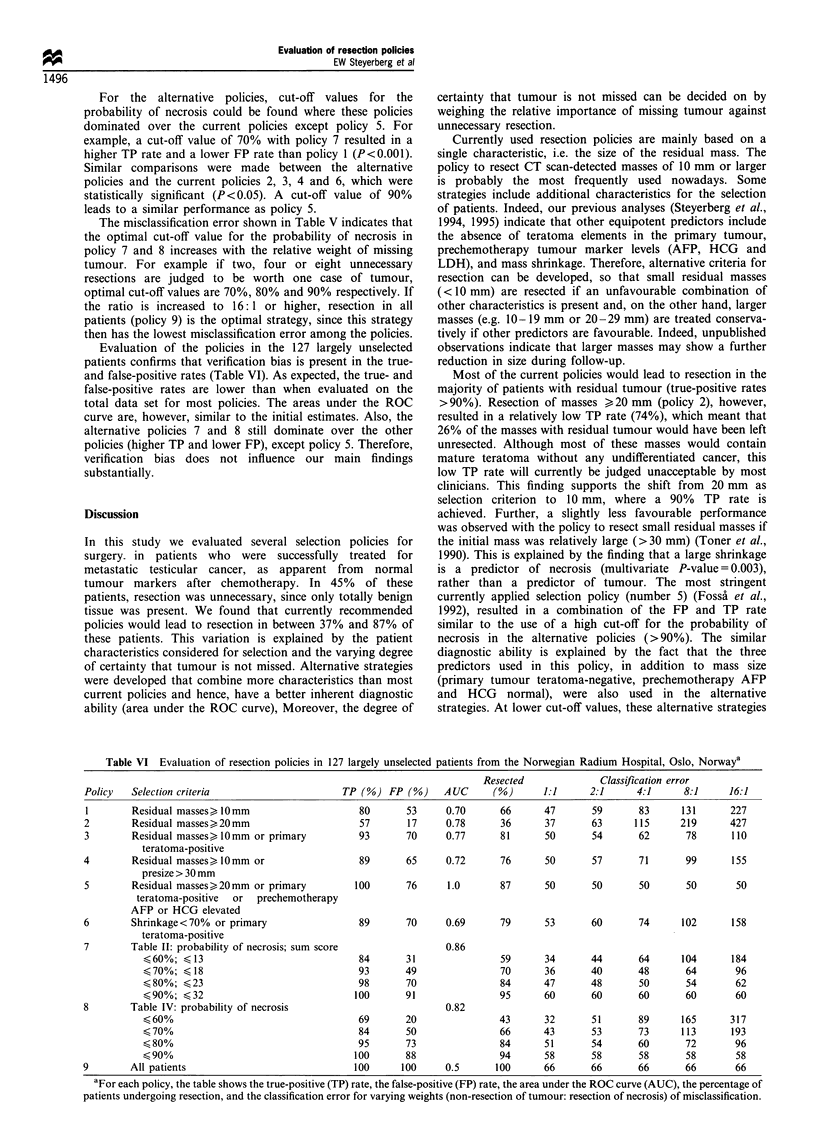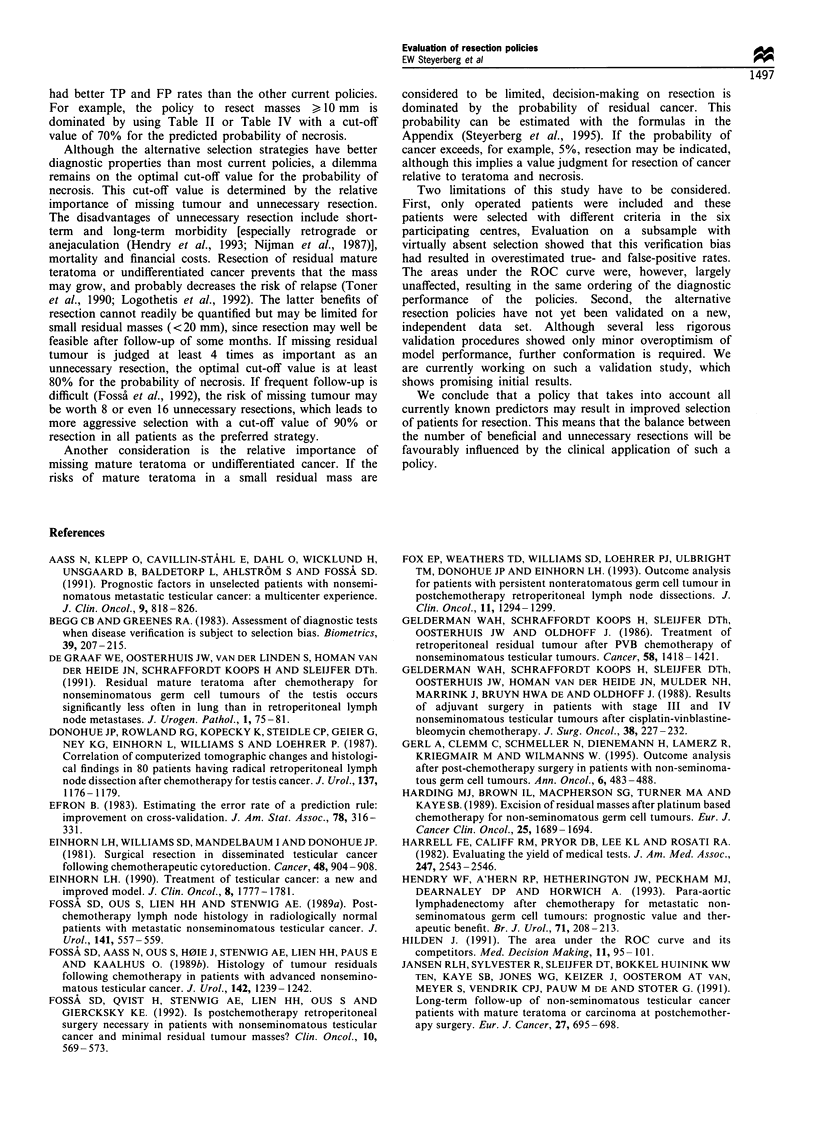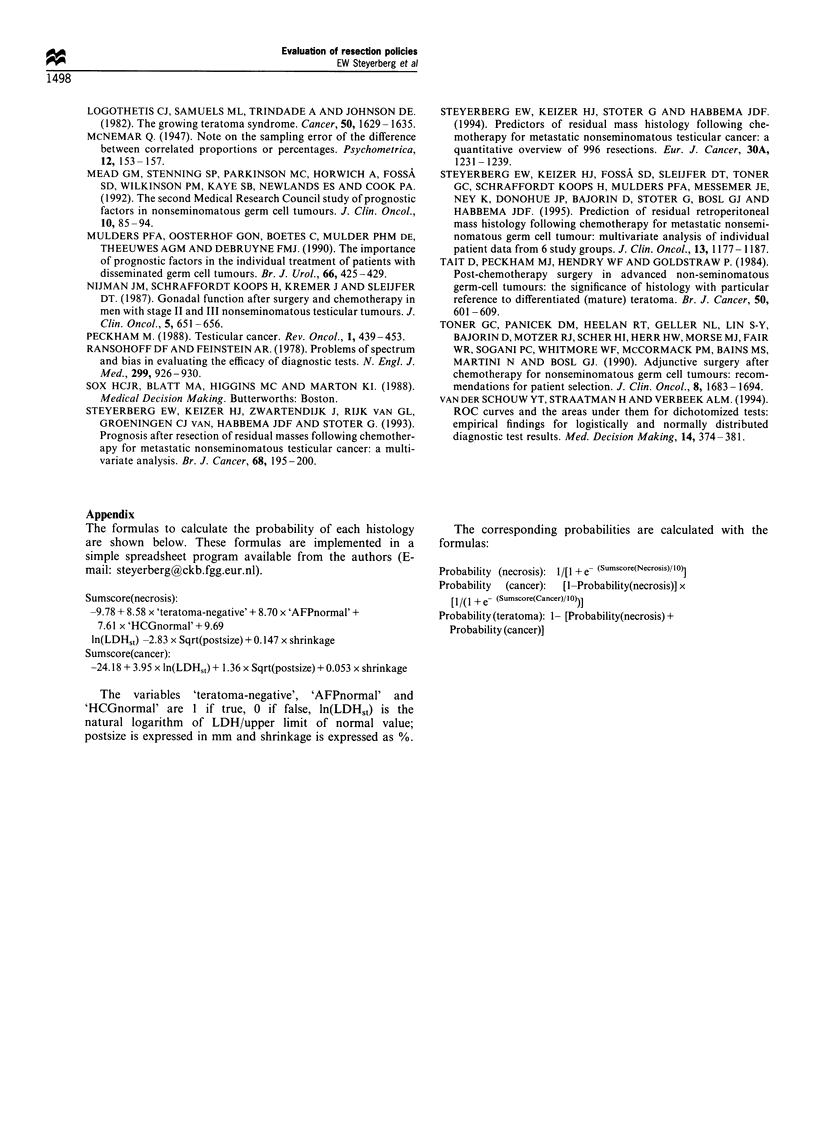# Resection of residual retroperitoneal masses in testicular cancer: evaluation and improvement of selection criteria. The ReHiT study group. Re-analysis of histology in testicular cancer.

**DOI:** 10.1038/bjc.1996.571

**Published:** 1996-11

**Authors:** E. W. Steyerberg, H. J. Keizer, S. D. Fosså, D. T. Sleijfer, D. F. Bajorin, J. P. Donohue, J. D. Habbema

**Affiliations:** Department of Public Health, Erasmus University, Rotterdam, The Netherlands.

## Abstract

Residual retroperitoneal masses may remain after chemotherapy for metastatic non-seminomatous testicular cancer, which harbour residual tumour or totally benign tissue (necrosis/fibrosis). These residual masses may be effectively removed by a surgical resection. We evaluated current selection criteria and tried to develop alternative criteria in a data set of 544 patients, who had retroperitoneal lymph node dissection of residual masses. Six resection policies were identified from the literature. Two alternative policies were developed with logistic regression analysis. Evaluation of the policies focused on the true-positive rate (resection in case of tumour), and the false-positive rate (resection in case of necrosis). It appeared that most current policies use the size of the residual mass (> or = 10 mm or > or = 20 mm) as the predominant selection criterion. This resulted in high true-positive rates (most > 90%), but false-positive rates between 37% and 87%. The alternative policies included five well-known predictors of necrosis in addition to residual mass size (primary tumour histology, prechemotherapy levels of the three tumour markers alphafetoprotein (AFP), human chorionic gonadotropin (HCG) and lactate dehydrogenase (LDH) and mass shrinkage during chemotherapy). This strategy resulted in improved true- and false-positive rates, even when categories of the predictors were simplified for practical application. We conclude that a simple statistical model, based on a limited number of patient characteristics, provides better guidelines for patient selection than those currently used in clinical practice.


British Journal of Cancer (1996) 74, 1492-1498
© 1996 Stockton Press All rights reserved 0007-0920/96 $12.00

### Resection of residual retroperitoneal masses in testicular cancer: evaluation and improvement of selection criteria

EW Steyerberg[1], HJ Keizer[2], SD Fosså[3], DT Sleijfer[4], DF Bajorin[5], JP Donohue[6] and JDF Habbema[1] for the ReHiT study group

[1]*Center for Clinical Decision Sciences, Department of Public Health, Erasmus University, Rotterdam, The Netherlands;* [2]*Department of Clinical Oncology, University Hospital, Leiden, The Netherlands;* [3]*Department of Medical Oncology and Radiotherapy, The Norwegian Radium Hospital, Oslo, Norway;* [4]*Department of Medical and Surgical Oncology, University Hospital, Groningen, The Netherlands;* [5]*Genitourinary Oncology Service, Division of Solid Tumour Oncology, Department of Medicine, Memorial Sloan-Kettering Cancer Center, New York, NY, USA;* [6]*Department of Urology, Indiana University School of Medicine, Indianapolis, Indiana, USA.*

**Summary** Residual retroperitoneal masses may remain after chemotherapy for metastatic non-seminomatous testicular cancer, which harbour residual tumour or totally benign tissue (necrosis/fibrosis). These residual masses may be effectively removed by a surgical resection. We evaluated current selection criteria and tried to develop alternative criteria in a data set of 544 patients, who had retroperitoneal lymph node dissection of residual masses. Six resection policies were identified from the literature. Two alternative policies were developed with logistic regression analysis. Evaluation of the policies focused on the true-positive rate (resection in case of tumour), and the false-positive rate (resection in case of necrosis). It appeared that most current policies use the size of the residual mass (⩾10 mm or ⩾20 mm) as the predominant selection criterion. This resulted in high true-positive rates (most >90%), but false-positive rates between 37% and 87%. The alternative policies included five well-known predictors of necrosis in addition to residual mass size (primary tumour histology, prechemotherapy levels of the three tumour markers alphafetoprotein (AFP), human chorionic gonadotropin (HCG) and lactate dehydrogenase (LDH) and mass shrinkage during chemotherapy). This strategy resulted in improved true- and false-positive rates, even when categories of the predictors were simplified for practical application. We conclude that a simple statistical model, based on a limited number of patient characteristics, provides better guidelines for patient selection than those currently used in clinical practice.

**Keywords:** testicular cancer; residual mass; resection

Testicular cancer is the most common malignancy among men in the age between 20 and 35 years. Fortunately, even metastatic disease can currently be cured in the majority (60–80%) of patients with non-seminomatous germ cell tumour, since the introduction of cisplatin-based chemotherapy (Peckham, 1988; Einhorn, 1990). After chemotherapy, surgical resection is a generally accepted treatment to remove residual retroperitoneal lymph node masses, since these masses still harbour residual tumour in about half of the patients. Alternatively, patients may be treated conservatively, which includes follow-up with regular blood tests and computerised tomography (CT) scans of the abdomen. A uniform approach to the selection of patients for resection is lacking (Toner *et al.*, 1990; Fosså *et al.*, 1992; Hendry *et al.*, 1993; Mulders *et al.*, 1990; Steyerberg *et al.*, 1993), and percentages of surgically treated patients vary between 20% (Mead *et al.*, 1992; Tait *et al.*, 1984) and 86% (Aass *et al.*, 1991). Therefore, several large cancer centres cooperated to evaluate the selection criteria for resection.

Correspondence: EW Steyerberg, Center for Clinical Decision Sciences, Ee 2091, Department of Public Health, Faculty of Medicine, Erasmus University, PO Box 1738, 3000 DR Rotterdam, The Netherlands

The ReHit (Re-analysis of histology in testicular cancer) study group consists of EW Steyerberg and Professor JDF Habbema, Erasmus University, Rotterdam; HJ Keizer, University Hospital, Leiden; DT Sleijfer and Professor H Schraffordt Koops; University Hospital, Groningen; PFA Mulders, University Hospital, Nijmegen; Professor G Stoter, Rotterdam Cancer Institute, The Netherlands; Professor SD Fosså; The Norwegian Radium Hospital, Oslo, Norway; GC Toner, Peter MacCallum Cancer Institute, Melbourne, Australia; DF Bajorin and Professor GJ Bosl, Memorial Sloan-Kettering Cancer Center, New York; JE Messemer, K Ney and Professor JP Donohue, Indiana University School of Medicine, Indianapolis, USA

Received 15 February 1996; revised 29 April 1996; accepted 15 May 1996

Resection of residual masses provides the histological diagnosis, which may be purely benign with necrotic and/or fibrotic remnants only (necrosis), or residual tumour (mature teratoma or undifferentiated cancer). In the case of cancer, two additional courses of chemotherapy are usually recommended (Einhorn *et al.*, 1981; Fox *et al.*, 1993). Although not proven, it may be assumed that this additional therapy reduces the risk of relapse, in addition to the resection itself. Resection of mature teratoma prevents growth of the residual mass (Logothetis *et al.*, 1982). In contrast, resection of benign masses has no therapeutic benefit. An ideal resection policy would, therefore, result in surgical removal of all masses with residual tumour (mature teratoma or cancer) and in a conservative treatment of all masses with necrosis.

Current selection policies were evaluated in an international data set from six study groups. A statistical model was developed from this same data set, using several well-known predictors of the histology of residual masses (Tait *et al.*, 1984; Donohue *et al.*, 1987; Gelderman *et al.*, 1988; Harding *et al.*, 1989; Toner *et al.*, 1990; Mulders *et al.*, 1990; Fosså *et al.*, 1992; Steyerberg *et al.*, 1994; Gerl *et al.*, 1995). Easy-to-use alternative selection criteria were based on this analysis and compared with the current policies.

### Patients and methods

### *Patients*

An international data set was collected, consisting of patients with metastatic non-seminomatous testicular cancer, including patients with pure seminoma and elevated levels of prechemotherapy tumour markers, who underwent resection of retroperitoneal residual masses after induction chemotherapy with cisplatin-based chemotherapy (Steyerberg *et al.*, 1995). Excluded were patients with elevated tumour markers

[alphafetoprotein (AFP) or human chorionic gonadotropin (HCG)] at the time of surgery, patients with extragonadal tumours, patients with pure seminoma and patients resected after relapse of tumour following initial chemotherapy.

Individual patient data included basic patient identification, histology at resection, and the following predictors: presence of teratoma elements in the primary tumour, prechemotherapy tumour marker levels [AFP, HCG, lactate dehydrogenase (LDH)], and pre- and post-chemotherapy mass size. Patients were included from Memorial Sloan-Kettering Cancer Center (MSKCC, $n=121$) (Toner *et al.*, 1990), Norwegian Radium Hospital (NRH, $n=127$) (Fosså *et al.*, 1989*a*,*b*, 1992; Aass *et al.*, 1991), Indiana University Hospital (IUH, $n=42$) (Donohue *et al.*, 1987), University Hospital Groningen (UHG, $n=137$) (Gelderman *et al.*, 1986, 1988; Nijman *et al.*, 1987; De Graaf *et al.*, 1991), and four other Dutch centres [University Hospitals of Nijmegen (Mulders *et al.*, 1990), Leiden, Amsterdam and Rotterdam (Steyerberg *et al.*, 1993): $n=117$]. Most European patients were treated according to trial protocols of the EORTC and MRC. In all centres, patients with residual abnormalities on radiological studies were recommended to undergo resection. Adherence to this recommendation was not evaluated in this study. In addition, patients with initial bulky retroperitoneal disease (diameter ⩾30 mm) were candidates for resection at MSKCC (Toner *et al.*, 1990), as well as UHG patients with teratoma elements in their primary tumour from 1988 onwards (Gelderman *et al.*, 1988). At NRH, resection was performed routinely in all patients with retroperitoneal lymph node enlargement at diagnosis (Aass *et al.*, 1991). The 42 patients included in this analysis from Indiana (IUH) all had a palpable prechemotherapy mass larger than 10 cm (Donohue *et al.*, 1987). This series thus represents a small part only of the experience at IUH with resection of residual masses. A total of 544 patients had all the required data available for analysis, of whom 245 (45%) had resection of necrosis only and 299 (55%) of residual tumour. Of these 299 patients, 68 had undifferentiated cancer (23%) and 231 had mature teratoma (77%). Patients were treated between 1975 and 1993, with a minority (11%) treated before 1981, and most between 1981 and 1985 (51%).

### *Methods*

Current resection policies were evaluated in the international data set. The probabilities of each residual histology (necrosis, mature teratoma, undifferentiated cancer) were calculated in masses that would be selected for resection and in masses that would be treated conservatively according to each policy. The policies were further evaluated as diagnostic tests, using the histology at resection as the gold standard diagnosis (Sox *et al.*, 1988). The true positive rate (or sensitivity) of a policy referred to the fraction of resected patients among those with residual tumour. The false-positive rate (or 1 minus specificity) referred to the fraction of patients who would undergo resection among the patients with necrosis. A perfect resection policy would have a true-positive rate of 100% and a false-positive rate of 0%. Areas under the receiver operating characteristic (ROC) curve were estimated to facilitate comparison of the diagnostic quality of the policies, assuming a logistic distribution of the data (Van der Schouw *et al.*, 1994). An area of 0.5 would arise if patients with and without residual tumour were equally likely to undergo resection. An area of 1.0 corresponds to a perfect policy.

Alternative resection criteria were developed with logistic regression analysis (SPSS/PC+ v5.01 software; SPSS Inc, Chicago, IL, USA, and SAS v6.04 software; SAS Institute Inc, Cary, NC, USA). The probability of necrosis was estimated for combinations of characteristics known before resection (predictors). A previous analysis of the data set showed that important predictors of necrosis were: the absence of teratoma elements in the primary tumour, prechemotherapy normal AFP, normal HCG, high LDH, a small post-chemotherapy mass size and a large shrinkage in size during chemotherapy (Steyerberg *et al.*, 1995). The latter three predictors were modelled as continuous variables, including transformations of post-chemotherapy size (square root) and prechemotherapy LDH (logarithmic). This model showed good results with extensive validation procedures, including bootstrapping (Efron, 1983) and leave-one-study-out evaluations. To facilitate application in clinical practice, we simplified the analysis by categorising the prechemotherapy LDH value (elevated *vs* normal according to the upper limit of the normal range for each centre, post-chemotherapy size (0–9 mm, 10–19 mm, 20–29 mm, 30–49 mm, ⩾50 mm) and shrinkage (reduction in maximum transversal diameter <0%, 0–69.9%, ⩾70%). Both for the original and for the simplified model, we calculated areas under the ROC curve (Harrell *et al.*, 1982). True- and false-positive rates were calculated with increasing cut-off values for the probability of necrosis.

### *Comparison of policies*

The diagnostic quality of the policies could be compared with the area under the ROC curve, with larger areas indicating better policies. A limitation of the area under the ROC curve is, however, that it does not consider the frequency of the outcome (necrosis/tumour at resection), nor the relative importance of misclassifications (Hilden, 1991). We, therefore, calculated a weighted classification error. The relative importance (or weight) of missing residual tumour was set as 1, 2, 4, 8 and 16 times that of unnecesary action. The weighted classification error was expressed as the number of unnecessary resections of necrosis and was calculated as: (number of unnecessary resections) + (weight × number of missed resections of tumour).

McNemar's test for paired observations was used for statistical comparisons between the policies (McNemar, 1947). Since the test assumes equal weights for false-positive and false-negative misclassifications, fair statistical comparisons could only be made if one policy dominated, i.e. had both a higher true- and a lower false-positive rate.

### *Verification bias*

In this analysis, data are only available from patients where the residual histology was verified by resection. These patients were selected from the total population of patients with normal tumour markers after chemotherapy according to the centre-specific selection policies. These policies had resulted in an average of 31% of the resections being performed in masses with a size of 0–10 mm (Steyerberg *et al.*, 1995). This selection may have led to a bias, labelled verification bias (Ransohoff and Feinstein, 1978; Begg and Greenes, 1983). This bias would lead to overestimated true- and false-positive rates, but to largely unbiased predicted probabilities of necrosis. Correction for verification bias in the international data set is difficult, since six different centres participated. Fortunately, in one centre resection was performed routinely (NRH, $n=127$) (Aass *et al.*, 1991), such that virtual absence of verification bias might be assumed here. This assumption was supported by the observation that 43% of the NRH resections had been performed in masses with a size of 0–10 mm. The policies were, therefore, also evaluated separately in these 127 patients.

### Results

Table I shows the current resection policies that were evaluated. The histological distribution is shown in masses that would be resected or treated conservatively according to each policy. The first policy (resection of all masses ⩾10 mm) has been widely applied in European centres (Mulders *et al.*, 1990; Jansen *et al.*, 1991; Steyerberg *et al.*, 1993). Masses

$\geq$10 mm are generally detected on CT scans, and this practice thus corresponds to resection if residual masses are detected on CT scans. It can be read from Table I that the probability of necrosis was 38% in masses $\geq$10 mm, in contrast to 72% in masses <10 mm. The second policy (resection of masses $\geq$20 mm) has been used especially in British centres (Tait *et al.*, 1984; Mead *et al.*, 1992; Hendry *et al.*, 1993). It would leave masses unresected with a low risk of undifferentiated cancer (4%), but a considerable risk of mature teratoma (30%). Policies 3 to 5 use one or more patient characteristics in addition to residual mass size. If resection is performed in all patients with a teratoma-positive tumour (policy 3, Gelderman *et al.*, 1988), the risk of leaving tumour unresected reduces to 23% (15% + 8%) compared with 28% with policy 1. Policy 4 (Toner *et al.*, 1990) leads to similar risk of missing residual tumour compared with policy 1 (30% *vs* 28%). Policy 5 (Fosså *et al.*, 1992) consists of resection in all patients, except a small subgroup with residual masses <20 mm and three favourable characteristics (primary tumour teratoma-negative and prechemotherapy AFP and HCG normal). This stringent practice does not guarantee that no tumour is missed, but the risk is low (6% + 6% = 12%). Policy 6 (Donohue *et al.*, 1987) consists of conservative treatment of patients with a shrinkage over 70% and a teratoma-negative primary tumour. Residual tumour was found in 24% (17% + 7%) of these patients.

*Alternative resection policies*

Alternative resection policies were based on statistical analysis of the international data set. The results of an analysis with continuous predictors are presented in Table II (Steyerberg *et al.*, 1995). The probability of necrosis corresponds to the sum score and can readily be calculated for individual patients. Exact formulas to calculate the probability of necrosis, mature teratoma and cancer are presented in the Appendix.

A simplified model used categories instead of the continuous predictors in the logistic regression original model. It was anticipated that the performance of this model would only be slightly worse than the original model, while the application in clinical practice would be facilitated. The categorised predictors as shown in Table III were analysed simultaneously with residual mass size. All five predictors had similar odds ratios (Table III: range 2.2–2.8). Therefore, a 'simple score' was constructed by counting the number of favourable characteristics.

Next, we used the two models (Table II and III) to derive alternative resection strategies. These alternative strategies use a cut-off value for the probability of necrosis. If the predicted probability of necrosis is lower than the cut-off value, resection is performed; if not, conservative treatment will follow. The choice of the cut-off values was based on the

**Table I** Resection policies and the histology of residual masses

| *Policy* | *Resection if* | *R/C*[a] | *Total* n=*544* *100%* | *Necrosis* n=*245* *45%* | *Teratoma* n=*231* *43%* | *Cancer* n=*68* *13%* |
|---|---|---|---|---|---|---|
| 1 | Residual masses $\geq$10 mm | R | 437 | 38% | 47% | 14% |
| | | C | 107 | 72% | 22% | 6% |
| 2 | Residual masses $\geq$20 mm | R | 313 | 29% | 52% | 19% |
| | | C | 231 | 67% | 30% | 4% |
| 3 | Residual masses $\geq$10 mm or primary tumour | R | 482 | 41% | 46% | 13% |
| | teratoma-positive | C | 62 | 77% | 15% | 8% |
| 4 | Residual masses $\geq$10 mm or prechemotherapy | R | 480 | 42% | 45% | 14% |
| | mass > 30 mm | C | 64 | 70% | 25% | 5% |
| 5 | Residual masses $\geq$20 mm or primary tumour | R | 508 | 42% | 45% | 13% |
| | teratoma-positive or prechemotherapy AFP/HCG elevated | C | 36 | 89% | 6% | 6% |
| 6 | Shrinkage in size < 70% or primary | R | 456 | 39% | 47% | 14% |
| | tumour teratoma-positive | C | 88 | 76% | 17% | 7% |

All patients had normal tumour markers AFP and HCG after chemotherapy for metastatic non-seminomatous testicular cancer. [a]R, patients fulfilling resection criteria; C, patients fulfilling conservative treatment criteria.

**Table II** Prognostic score chart to estimate the probability of necrosis in residual retroperitoneal masses

| *Predictor* | *Value* | | | | | | | *Score* |
|---|---|---|---|---|---|---|---|---|
| Primary tumour histology | | | | | | | | |
| Teratoma-negative | +9 | | | | | | | ...... |
| Prechemotherapy markers | | | | | | | | |
| Normal AFP | +9 | | | | | | | ...... |
| Normal HCG | +8 | | | | | | | ...... |
| LDH/normal value[a] | 0.6 | 0.8 | 1.0 | 1.5 | 2.0 | 3.0 | 4.5 | |
| Score | −5 | −2 | 0 | +4 | +7 | +11 | +15 | ...... |
| Postchemotherapy mass size | | | | | | | | |
| Transversal diameter (mm)[a] | 2[b] | 5 | 10 | 20 | 30 | 50 | 100 | |
| Score | −4 | −6 | −9 | −13 | −16 | −20 | −28 | ...... |
| Shrinkage | | | | | | | | |
| 100 × (presize-postsize)/presize[a] | −50 | 0 | 50 | 75 | 100 | | | |
| Score | −7 | 0 | +7 | +11 | +15 | | | ...... |
| Estimate individual probability of necrosis | | | | | Sum score (add) | | | ...... |
| Sum score | 10 | 15 | 20 | 25 | 30 | 35 | 40 | |
| Probability (%) | 51 | 63 | 74 | 82 | 88 | 93 | 95 | |

[a]Continuous variables; scores for intermediate values can be estimated with linear interpolation. [b]If no mass is detectable on the post-chemotherapy CT scan, a size of 2 mm is assumed.

observed probabilities with the current policies, which apply cut-off values implicity. With 60% and 90% as extremes of the probability of necrosis, two areas with a clear treatment advice evolve. If the probability of necrosis is less than 60%, resection should follow; if the probability exceeds 90%, conservative treatment is advised. In between is a grey area, where the decision to resect a residual mass depends on the cut-off value applied (60%, 70%, 80% or 90%). Table IV shows the probability of necrosis according to the simplified logistic regression model. It can, for instance, be read that the probability of necrosis is less than 60% in patients with a residual mass ⩾50 mm, in patients with a mass that increased during chemotherapy, in patients with a low score (0 or 1 point), in patients with a mass of 20–29 mm and a score of 2 points, and in patients with a mass of 30–49 mm and a score of 3 points.

**Table III** Categorised predictors of necrosis in addition to residual mass size

| *Characteristic* | *OR* | *95% CI* | *Score* |
|---|---|---|---|
| Primary tumour teratoma-negative | 2.7 | 1.8–4.2 | 0/1 |
| Prechemotherapy AFP normal | 2.4 | 1.5–3.9 | 0/1 |
| Prechemotherapy HCG normal | 2.2 | 1.4–3.4 | 0/1 |
| Prechemotherapy LDH elevated | 2.8 | 1.6–4.7 | 0/1 |
| Shrinkage in mass⩾70% | 2.2 | 1.3–3.9 | 0/1 |
| | | Simple score | 0–5[+] |

Odds ratios and 95% confidence intervals were calculated with logistic regression analysis ($n=544$).

*Evaluation of policies*

Table V shows the results of the evaluation of the current policies, the alternative policies, and the extreme policy of resection in all patients. The true-positive (TP) rate of the current policies (except policy 2) exceeds 90%. This means that over 90% of the patients with residual tumour would be resected with these policies and that less than 10% of the masses with tumour would be missed. The false-positive (FP) rate varies between 37% and 87%, which means that a large proportion of the patients with necrosis would undergo resection unnecessarily. Policy 2 is remarkable, as both the TP and FP rate are relatively low (74% and 37%). For the alternative policies (7 and 8), it is clear that an increase of the cut-off values for the probability of necrosis, leads to a larger fraction of resected patients and to higher TP and FP rates. Thus, the higher the required probability of necrosis for conservative treatment, the lower the risk of missing tumour, but the higher the risk of unnecessary resection. The diagnostic performance of the policies was further compared by the areas under the ROC curve. The performance of policies 1, 2, 3, 4 and 6 was more or less similar (area 0.72, 0.74, 0.75, 0.69 and 0.75). Policy 5 had a better diagnostic ability (area 0.84), similar to the alternative resection policies (7 and 8).

**Table IV** Probability (%) of necrosis is according to combinations of the simple score (Table III) and residual mass size

| *Mass size (mm)* | *Simple score* 0 | *1* | *2* | *3* | *4* | *5* |
|---|---|---|---|---|---|---|
| 0–9 | ⩽60 | ⩽60 | >60 | >70 | >80 | >90 |
| 10–19 | ⩽60 | ⩽60 | >60 | >70 | >80 | >90 |
| 20–29 | ⩽60 | ⩽60 | ⩽60 | >60 | >80 | >90 |
| 30–49 | ⩽60 | ⩽60 | ⩽60 | ⩽60 | >70 | >80 |
| ⩾50 or increased mass | ⩽60 | ⩽60 | ⩽60 | ⩽60 | ⩽60 | ⩽60 |

**Table V** Evaluation of resection policies in the 544 patients in the international data set[a]

| *Policy* | *Selection criteria* | *TP (%)* | *FP (%)* | *AUC* | *Resected (%)* | *Classification error* 1:1 | *2:1* | *4:1* | *8:1* | *16:1* |
|---|---|---|---|---|---|---|---|---|---|---|
| 1 | Residual masses⩾10 mm | 90 | 69 | 0.72 | 80 | 198 | 228 | 288 | 408 | 648 |
| 2 | Residual masses⩾20 mm | 74 | 37 | 0.74 | 58 | 168 | 245 | 399 | 707 | 1323 |
| 3 | Residual masses⩾10 mm or primary teratoma-positive | 95 | 80 | 0.75 | 89 | 211 | 225 | 253 | 309 | 421 |
| 4 | Residual masses⩾10 mm or presize>30 mm | 94 | 82 | 0.69 | 88 | 219 | 238 | 276 | 352 | 504 |
| 5 | Residual masses⩾20 mm or primary teratoma-positive or prechemotherapy AFP or HCG elevated | 98.7 | 87 | 0.84 | 93 | 217 | 221 | 229 | 245 | 277 |
| 6 | Shrinkage<70% or primary teratoma-positive | 93 | 73 | 0.75 | 84 | 199 | 220 | 262 | 346 | 514 |
| 7 | Table II: probability of necrosis; sum score | | | 0.84 | | | | | | |
| | ⩽60%; ⩽13 | 85 | 38 | | 64 | 136 | 180 | 268 | 444 | 796 |
| | ⩽70%; ⩽18 | 92 | 52 | | 74 | 153 | 178 | 228 | 328 | 528 |
| | ⩽80%; ⩽23 | 96 | 73 | | 86 | 190 | 201 | 223 | 267 | 355 |
| | ⩽90%; ⩽32 | 98.7 | 89 | | 94 | 222 | 226 | 234 | 250 | 282 |
| 8 | Table IV: probability of necrosis | | | 0.82 | | | | | | |
| | ⩽60% | 79 | 30 | | 57 | 138 | 202 | 330 | 586 | 1098 |
| | ⩽70% | 91 | 55 | | 74 | 162 | 190 | 246 | 358 | 582 |
| | ⩽80% | 97 | 76 | | 88 | 197 | 207 | 227 | 267 | 347 |
| | ⩽90% | 99.7 | 94 | | 97 | 231 | 232 | 234 | 238 | 246 |
| 9 | All patients | 100 | 100 | 0.5 | 100 | 245 | 245 | 245 | 245 | 245 |

[a]For each policy, the table shows the true-positive (TP) rate, the false-positive (FP) rate, the area under the ROC curve (AUC), the percentage of patients undergoing resection, and the classification error for varying weights (non-resection of tumour: resection of necrosis) of misclassification.

For the alternative policies, cut-off values for the probability of necrosis could be found where these policies dominated over the current policies except policy 5. For example, a cut-off value of 70% with policy 7 resulted in a higher TP rate and a lower FP rate than policy 1 ($P<0.001$). Similar comparisons were made between the alternative policies and the current policies 2, 3, 4 and 6, which were statistically significant ($P<0.05$). A cut-off value of 90% leads to a similar performance as policy 5.

The misclassification error shown in Table V indicates that the optimal cut-off value for the probability of necrosis in policy 7 and 8 increases with the relative weight of missing tumour. For example if two, four or eight unnecessary resections are judged to be worth one case of tumour, optimal cut-off values are 70%, 80% and 90% respectively. If the ratio is increased to 16:1 or higher, resection in all patients (policy 9) is the optimal strategy, since this strategy then has the lowest misclassification error among the policies.

Evaluation of the policies in the 127 largely unselected patients confirms that verification bias is present in the true- and false-positive rates (Table VI). As expected, the true- and false-positive rates are lower than when evaluated on the total data set for most policies. The areas under the ROC curve are, however, similar to the initial estimates. Also, the alternative policies 7 and 8 still dominate over the other policies (higher TP and lower FP), except policy 5. Therefore, verification bias does not influence our main findings substantially.

### Discussion

In this study we evaluated several selection policies for surgery in patients who were successfully treated for metastatic testicular cancer, as apparent from normal tumour markers after chemotherapy. In 45% of these patients, resection was unnecessary, since only totally benign tissue was present. We found that currently recommended policies would lead to resection in between 37% and 87% of these patients. This variation is explained by the patient characteristics considered for selection and the varying degree of certainty that tumour is not missed. Alternative strategies were developed that combine more characteristics than most current policies and hence, have a better inherent diagnostic ability (area under the ROC curve), Moreover, the degree of certainty that tumour is not missed can be decided on by weighing the relative importance of missing tumour against unnecessary resection.

Currently used resection policies are mainly based on a single characteristic, i.e. the size of the residual mass. The policy to resect CT scan-detected masses of 10 mm or larger is probably the most frequently used nowadays. Some strategies include additional characteristics for the selection of patients. Indeed, our previous analyses (Steyerberg *et al.*, 1994, 1995) indicate that other equipotent predictors include the absence of teratoma elements in the primary tumour, prechemotherapy tumour marker levels (AFP, HCG and LDH), and mass shrinkage. Therefore, alternative criteria for resection can be developed, so that small residual masses (<10 mm) are resected if an unfavourable combination of other characteristics is present and, on the other hand, larger masses (e.g. 10–19 mm or 20–29 mm) are treated conservatively if other predictors are favourable. Indeed, unpublished observations indicate that larger masses may show a further reduction in size during follow-up.

Most of the current policies would lead to resection in the majority of patients with residual tumour (true-positive rates >90%). Resection of masses ⩾20 mm (policy 2), however, resulted in a relatively low TP rate (74%), which meant that 26% of the masses with residual tumour would have been left unresected. Although most of these masses would contain mature teratoma without any undifferentiated cancer, this low TP rate will currently be judged unacceptable by most clinicians. This finding supports the shift from 20 mm as selection criterion to 10 mm, where a 90% TP rate is achieved. Further, a slightly less favourable performance was observed with the policy to resect small residual masses if the initial mass was relatively large (>30 mm) (Toner *et al.*, 1990). This is explained by the finding that a large shrinkage is a predictor of necrosis (multivariate $P$-value = 0.003), rather than a predictor of tumour. The most stringent currently applied selection policy (number 5) (Fosså *et al.*, 1992), resulted in a combination of the FP and TP rate similar to the use of a high cut-off for the probability of necrosis in the alternative policies (>90%). The similar diagnostic ability is explained by the fact that the three predictors used in this policy, in addition to mass size (primary tumour teratoma-negative, prechemotherapy AFP and HCG normal), were also used in the alternative strategies. At lower cut-off values, these alternative strategies

**Table VI** Evaluation of resection policies in 127 largely unselected patients from the Norwegian Radium Hospital, Oslo, Norway[a]

| Policy | Selection criteria | TP (%) | FP (%) | AUC | Resected (%) | Classification error 1:1 | 2:1 | 4:1 | 8:1 | 16:1 |
|---|---|---|---|---|---|---|---|---|---|---|
| 1 | Residual masses ⩾ 10 mm | 80 | 53 | 0.70 | 66 | 47 | 59 | 83 | 131 | 227 |
| 2 | Residual masses ⩾ 20 mm | 57 | 17 | 0.78 | 36 | 37 | 63 | 115 | 219 | 427 |
| 3 | Residual masses ⩾ 10 mm or primary teratoma-positive | 93 | 70 | 0.77 | 81 | 50 | 54 | 62 | 78 | 110 |
| 4 | Residual masses ⩾ 10 mm or presize > 30 mm | 89 | 65 | 0.72 | 76 | 50 | 57 | 71 | 99 | 155 |
| 5 | Residual masses ⩾ 20 mm or primary teratoma-positive or prechemotherapy AFP or HCG elevated | 100 | 76 | 1.0 | 87 | 50 | 50 | 50 | 50 | 50 |
| 6 | Shrinkage < 70% or primary teratoma-positive | 89 | 70 | 0.69 | 79 | 53 | 60 | 74 | 102 | 158 |
| 7 | Table II: probability of necrosis; sum score | | | 0.86 | | | | | | |
| | ⩽60%; ⩽13 | 84 | 31 | | 59 | 34 | 44 | 64 | 104 | 184 |
| | ⩽70%; ⩽18 | 93 | 49 | | 70 | 36 | 40 | 48 | 64 | 96 |
| | ⩽80%; ⩽23 | 98 | 70 | | 84 | 47 | 48 | 50 | 54 | 62 |
| | ⩽90%; ⩽32 | 100 | 91 | | 95 | 60 | 60 | 60 | 60 | 60 |
| 8 | Table IV: probability of necrosis | | | 0.82 | | | | | | |
| | ⩽60% | 69 | 20 | | 43 | 32 | 51 | 89 | 165 | 317 |
| | ⩽70% | 84 | 50 | | 66 | 43 | 53 | 73 | 113 | 193 |
| | ⩽80% | 95 | 73 | | 84 | 51 | 54 | 60 | 72 | 96 |
| | ⩽90% | 100 | 88 | | 94 | 58 | 58 | 58 | 58 | 58 |
| 9 | All patients | 100 | 100 | 0.5 | 100 | 66 | 66 | 66 | 66 | 66 |

[a]For each policy, the table shows the true-positive (TP) rate, the false-positive (FP) rate, the area under the ROC curve (AUC), the percentage of patients undergoing resection, and the classification error for varying weights (non-resection of tumour: resection of necrosis) of misclassification.

had better TP and FP rates than the other current policies. For example, the policy to resect masses ⩾10 mm is dominated by using Table II or Table IV with a cut-off value of 70% for the predicted probability of necrosis.

Although the alternative selection strategies have better diagnostic properties than most current policies, a dilemma remains on the optimal cut-off value for the probability of necrosis. This cut-off value is determined by the relative importance of missing tumour and unnecessary resection. The disadvantages of unnecessary resection include short-term and long-term morbidity [especially retrograde or anejaculation (Hendry *et al.*, 1993; Nijman *et al.*, 1987)], mortality and financial costs. Resection of residual mature teratoma or undifferentiated cancer prevents that the mass may grow, and probably decreases the risk of relapse (Toner *et al.*, 1990; Logothetis *et al.*, 1992). The latter benefits of resection cannot readily be quantified but may be limited for small residual masses (<20 mm), since resection may well be feasible after follow-up of some months. If missing residual tumour is judged at least 4 times as important as an unnecessary resection, the optimal cut-off value is at least 80% for the probability of necrosis. If frequent follow-up is difficult (Fosså *et al.*, 1992), the risk of missing tumour may be worth 8 or even 16 unnecessary resections, which leads to more aggressive selection with a cut-off value of 90% or resection in all patients as the preferred strategy.

Another consideration is the relative importance of missing mature teratoma or undifferentiated cancer. If the risks of mature teratoma in a small residual mass are considered to be limited, decision-making on resection is dominated by the probability of residual cancer. This probability can be estimated with the formulas in the Appendix (Steyerberg *et al.*, 1995). If the probability of cancer exceeds, for example, 5%, resection may be indicated, although this implies a value judgment for resection of cancer relative to teratoma and necrosis.

Two limitations of this study have to be considered. First, only operated patients were included and these patients were selected with different criteria in the six participating centres, Evaluation on a subsample with virtually absent selection showed that this verification bias had resulted in overestimated true- and false-positive rates. The areas under the ROC curve were, however, largely unaffected, resulting in the same ordering of the diagnostic performance of the policies. Second, the alternative resection policies have not yet been validated on a new, independent data set. Although several less rigorous validation procedures showed only minor overoptimism of model performance, further conformation is required. We are currently working on such a validation study, which shows promising initial results.

We conclude that a policy that takes into account all currently known predictors may result in improved selection of patients for resection. This means that the balance between the number of beneficial and unnecessary resections will be favourably influenced by the clinical application of such a policy.

### References

AASS N, KLEPP O, CAVILLIN-STÅHL E, DAHL O, WICKLUND H, UNSGAARD B, BALDETORP L, AHLSTRÖM S AND FOSSÅ SD. (1991). Prognostic factors in unselected patients with nonseminomatous metastatic testicular cancer: a multicenter experience. *J. Clin. Oncol.*, **9**, 818–826.

BEGG CB AND GREENES RA. (1983). Assessment of diagnostic tests when disease verification is subject to selection bias. *Biometrics*, **39**, 207–215.

DE GRAAF WE, OOSTERHUIS JW, VAN DER LINDEN S, HOMAN VAN DER HEIDE JN, SCHRAFFORDT KOOPS H AND SLEIJFER DTh. (1991). Residual mature teratoma after chemotherapy for nonseminomatous germ cell tumours of the testis occurs significantly less often in lung than in retroperitoneal lymph node metastases. *J. Urogen. Pathol.*, **1**, 75–81.

DONOHUE JP, ROWLAND RG, KOPECKY K, STEIDLE CP, GEIER G, NEY KG, EINHORN L, WILLIAMS S AND LOEHRER P. (1987). Correlation of computerized tomographic changes and histological findings in 80 patients having radical retroperitoneal lymph node dissection after chemotherapy for testis cancer. *J. Urol.*, **137**, 1176–1179.

EFRON B. (1983). Estimating the error rate of a prediction rule: improvement on cross-validation. *J. Am. Stat. Assoc.*, **78**, 316–331.

EINHORN LH, WILLIAMS SD, MANDELBAUM I AND DONOHUE JP. (1981). Surgical resection in disseminated testicular cancer following chemotherapeutic cytoreduction. *Cancer*, **48**, 904–908.

EINHORN LH. (1990). Treatment of testicular cancer: a new and improved model. *J. Clin. Oncol.*, **8**, 1777–1781.

FOSSÅ SD, OUS S, LIEN HH AND STENWIG AE. (1989*a*). Post-chemotherapy lymph node histology in radiologically normal patients with metastatic nonseminomatous testicular cancer. *J. Urol.*, **141**, 557–559.

FOSSÅ SD, AASS N, OUS S, HØIE J, STENWIG AE, LIEN HH, PAUS E AND KAALHUS O. (1989*b*). Histology of tumour residuals following chemotherapy in patients with advanced nonseminomatous testicular cancer. *J. Urol.*, **142**, 1239–1242.

FOSSÅ SD, QVIST H, STENWIG AE, LIEN HH, OUS S AND GIERCKSKY KE. (1992). Is postchemotherapy retroperitoneal surgery necessary in patients with nonseminomatous testicular cancer and minimal residual tumour masses? *Clin. Oncol.*, **10**, 569–573.

FOX EP, WEATHERS TD, WILLIAMS SD, LOEHRER PJ, ULBRIGHT TM, DONOHUE JP AND EINHORN LH. (1993). Outcome analysis for patients with persistent nonteratomatous germ cell tumour in postchemotherapy retroperitoneal lymph node dissections. *J. Clin. Oncol.*, **11**, 1294–1299.

GELDERMAN WAH, SCHRAFFORDT KOOPS H, SLEIJFER DTh, OOSTERHUIS JW AND OLDHOFF J. (1986). Treatment of retroperitoneal residual tumour after PVB chemotherapy of nonseminomatous testicular tumours. *Cancer*, **58**, 1418–1421.

GELDERMAN WAH, SCHRAFFORDT KOOPS H, SLEIJFER DTh, OOSTERHUIS JW, HOMAN VAN DER HEIDE JN, MULDER NH, MARRINK J, BRUYN HWA DE AND OLDHOFF J. (1988). Results of adjuvant surgery in patients with stage III and IV nonseminomatous testicular tumours after cisplatin-vinblastine-bleomycin chemotherapy. *J. Surg. Oncol.*, **38**, 227–232.

GERL A, CLEMM C, SCHMELLER N, DIENEMANN H, LAMERZ R, KRIEGMAIR M AND WILMANNS W. (1995). Outcome analysis after post-chemotherapy surgery in patients with non-seminomatous germ cell tumours. *Ann. Oncol.*, **6**, 483–488.

HARDING MJ, BROWN IL, MACPHERSON SG, TURNER MA AND KAYE SB. (1989). Excision of residual masses after platinum based chemotherapy for non-seminomatous germ cell tumours. *Eur. J. Cancer Clin. Oncol.*, **25**, 1689–1694.

HARRELL FE, CALIFF RM, PRYOR DB, LEE KL AND ROSATI RA. (1982). Evaluating the yield of medical tests. *J. Am. Med. Assoc.*, **247**, 2543–2546.

HENDRY WF, A'HERN RP, HETHERINGTON JW, PECKHAM MJ, DEARNALEY DP AND HORWICH A. (1993). Para-aortic lymphadenectomy after chemotherapy for metastatic non-seminomatous germ cell tumours: prognostic value and therapeutic benefit. *Br. J. Urol.*, **71**, 208–213.

HILDEN J. (1991). The area under the ROC curve and its competitors. *Med. Decision Making*, **11**, 95–101.

JANSEN RLH, SYLVESTER R, SLEIJFER DT, BOKKEL HUININK WW TEN, KAYE SB, JONES WG, KEIZER J, OOSTEROM AT VAN, MEYER S, VENDRIK CPJ, PAUW M DE AND STOTER G. (1991). Long-term follow-up of non-seminomatous testicular cancer patients with mature teratoma or carcinoma at postchemotherapy surgery. *Eur. J. Cancer*, **27**, 695–698.

LOGOTHETIS CJ, SAMUELS ML, TRINDADE A AND JOHNSON DE. (1982). The growing teratoma syndrome. *Cancer*, **50**, 1629–1635.

MCNEMAR Q. (1947). Note on the sampling error of the difference between correlated proportions or percentages. *Psychometrica*, **12**, 153–157.

MEAD GM, STENNING SP, PARKINSON MC, HORWICH A, FOSSÅ SD, WILKINSON PM, KAYE SB, NEWLANDS ES AND COOK PA. (1992). The second Medical Research Council study of prognostic factors in nonseminomatous germ cell tumours. *J. Clin. Oncol.*, **10**, 85–94.

MULDERS PFA, OOSTERHOF GON, BOETES C, MULDER PHM DE, THEEUWES AGM AND DEBRUYNE FMJ. (1990). The importance of prognostic factors in the individual treatment of patients with disseminated germ cell tumours. *Br. J. Urol.*, **66**, 425–429.

NIJMAN JM, SCHRAFFORDT KOOPS H, KREMER J AND SLEIJFER DT. (1987). Gonadal function after surgery and chemotherapy in men with stage II and III nonseminomatous testicular tumours. *J. Clin. Oncol.*, **5**, 651–656.

PECKHAM M. (1988). Testicular cancer. *Rev. Oncol.*, **1**, 439–453.

RANSOHOFF DF AND FEINSTEIN AR. (1978). Problems of spectrum and bias in evaluating the efficacy of diagnostic tests. *N. Engl. J. Med.*, **299**, 926–930.

SOX HCJR, BLATT MA, HIGGINS MC AND MARTON KI. (1988). *Medical Decision Making*. Butterworths: Boston.

STEYERBERG EW, KEIZER HJ, ZWARTENDIJK J, RIJK VAN GL, GROENINGEN CJ VAN, HABBEMA JDF AND STOTER G. (1993). Prognosis after resection of residual masses following chemotherapy for metastatic nonseminomatous testicular cancer: a multivariate analysis. *Br. J. Cancer*, **68**, 195–200.

STEYERBERG EW, KEIZER HJ, STOTER G AND HABBEMA JDF. (1994). Predictors of residual mass histology following chemotherapy for metastatic nonseminomatous testicular cancer: a quantitative overview of 996 resections. *Eur. J. Cancer*, **30A**, 1231–1239.

STEYERBERG EW, KEIZER HJ, FOSSÅ SD, SLEIJFER DT, TONER GC, SCHRAFFORDT KOOPS H, MULDERS PFA, MESSEMER JE, NEY K, DONOHUE JP, BAJORIN D, STOTER G, BOSL GJ AND HABBEMA JDF. (1995). Prediction of residual retroperitoneal mass histology following chemotherapy for metastatic nonseminomatous germ cell tumour: multivariate analysis of individual patient data from 6 study groups. *J. Clin. Oncol.*, **13**, 1177–1187.

TAIT D, PECKHAM MJ, HENDRY WF AND GOLDSTRAW P. (1984). Post-chemotherapy surgery in advanced non-seminomatous germ-cell tumours: the significance of histology with particular reference to differentiated (mature) teratoma. *Br. J. Cancer*, **50**, 601–609.

TONER GC, PANICEK DM, HEELAN RT, GELLER NL, LIN S-Y, BAJORIN D, MOTZER RJ, SCHER HI, HERR HW, MORSE MJ, FAIR WR, SOGANI PC, WHITMORE WF, MCCORMACK PM, BAINS MS, MARTINI N AND BOSL GJ. (1990). Adjunctive surgery after chemotherapy for nonseminomatous germ cell tumours: recommendations for patient selection. *J. Clin. Oncol.*, **8**, 1683–1694.

VAN DER SCHOUW YT, STRAATMAN H AND VERBEEK ALM. (1994). ROC curves and the areas under them for dichotomized tests: empirical findings for logistically and normally distributed diagnostic test results. *Med. Decision Making*, **14**, 374–381.

### Appendix

The formulas to calculate the probability of each histology are shown below. These formulas are implemented in a simple spreadsheet program available from the authors (E-mail: steyerberg@ckb.fgg.eur.nl).

Sumscore(necrosis):

$-9.78 + 8.58 \times$ 'teratoma-negative' $+ 8.70 \times$ 'AFPnormal' $+$

$7.61 \times$ 'HCGnormal' $+ 9.69$

$\ln(LDH_{st})$ $-2.83 \times \mathrm{Sqrt}(\text{postsize}) + 0.147 \times \text{shrinkage}$

Sumscore(cancer):

$-24.18 + 3.95 \times \ln(LDH_{st}) + 1.36 \times \mathrm{Sqrt}(\text{postsize}) + 0.053 \times \text{shrinkage}$

The variables 'teratoma-negative', 'AFPnormal' and 'HCGnormal' are 1 if true, 0 if false, $\ln(LDH_{st})$ is the natural logarithm of LDH/upper limit of normal value; postsize is expressed in mm and shrinkage is expressed as %.

The corresponding probabilities are calculated with the formulas:

Probability (necrosis): $1/[1 + e^{-(\text{Sumscore(Necrosis)}/10)}]$

Probability (cancer): $[1 - \text{Probability(necrosis)}] \times [1/(1 + e^{-(\text{Sumscore(Cancer)}/10)})]$

Probability (teratoma): 1– [Probability(necrosis) + Probability (cancer)]